# Synergistic Effects of Polystyrene Nanoplastics and Cadmium on the Metabolic Processes and Their Accumulation in Hydroponically Grown Lettuce ()

**DOI:** 10.1021/acs.jafc.5c03215

**Published:** 2025-06-24

**Authors:** Michael Taylor Bryant, Lorenzo Rossi, Ruipu Mu, Zhenyu Cao, Xingmao Ma

**Affiliations:** 1 Department of Civil and Environmental Engineering, 14736 Texas A&M University, College Station, Texas 77843, United States; 2 Department of Horticultural Sciences, 14736 Texas A&M University, College Station, Texas 77843, United States; 3 Basic Sciences Department, College of Arts and Sciences, University of Health Sciences and Pharmacy in St. Louis, St. Louis, Missouri 63110, United States

**Keywords:** heavy metals, plastic pollution, metabolic response, oxidative stress

## Abstract

Plastic contamination in agricultural systems is an emerging concern. While current research suggests low direct toxicity, the consequences from interactions between nanoplastics and copresent contaminants are poorly understood. In this study, the synergistic effects of cadmium (Cd) and polystyrene nanoplastics (PS NP) on the growth and physiological responses of hydroponically grown (lettuce) were examined. Coexposure significantly increased the accumulation of Cd and PS NP by 61 and 67% in lettuce shoots compared with single-contaminant exposure. Metabolomic analysis showed that joint exposure induced an increase in glutathione and flavonoid-like compounds, suggesting an energy-intensive oxidative stress response. In addition, coexposure appeared to promote adventitious root formation, as evidenced by an increased abundance of metabolites linked to nitric oxide signaling. These findings suggest that the projected increase in PS NP in agricultural environments could exacerbate Cd uptake in food crops, potentially increasing human dietary exposure to heavy metals.

## Graphical abstract

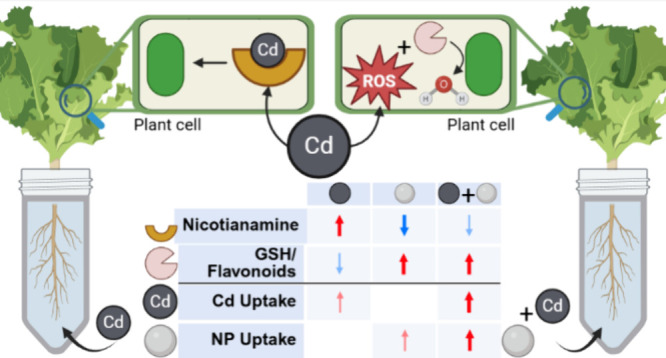

## 1 Introduction

Since the expansion of commercial plastic production in the 1960s, annual global output has soared to hundreds of millions of tons.^1^ Renowned for their chemical stability, plastics persist in the environment after disposal.^2^ However, plastics are not perfectly stable. Over time, they degrade into microplastics (MP, <5 mm) and nanoplastics (NP, <1 μm) through photo-oxidation,^3^ mechanical abrasion,^4^ and other weathering processes. The unchecked disposal of larger plastic debris has led to the widespread environmental presence of these micro- and nanoplastics (MNPs).^5,6^ Consequently, the potential negative impacts of MNP on ecosystems and biological systems, including human health, have garnered increasing research attention.^7–10^

One notable source of MNP exposure to humans is via food crop consumption.^11^ However, it was not until 2019 that definitive evidence of MNP accumulation and transport in plant tissues was reported,^12^ raising concerns about their broader impacts on food systems. Earlier studies reported reduced photosynthetic capacity,^12^ diminished carbon fixation, and decreased yield in several important food crops in addition to elevated accumulation in plant tissues.^13^ Other studies showed that MNPs decreased the accumulation of essential elements such as iron (Fe) and zinc (Zn), as well as essential amino acids like methionine and glutamine in edible tissues,^14^ potentially impairing plant growth and compromising both yield and nutritional quality.

However, MNPs are not the only environmental contaminants of concern. More recent studies have explored the combined effects of MNPs and co-occurring pollutants on plant stress responses and contaminant uptake.^15,16^ Heavy metals, a class of widespread agricultural pollutants, have long been recognized as a significant concern.^17^ Cadmium (Cd), a heavy metal naturally present in the environment^18^ but with elevated concentrations in some agricultural soils, has garnered research attention because of its phytotoxicity.^19^ For example, Cd was shown to induce oxidative stress,^20^ disrupt cellular functions and interfere with metabolic processes,^21^ and ultimately reduce crop yield.^22^

In environmental settings, these two contaminants may be copresent. Thus, coexposure studies are valuable to understanding their potential synergistic effects. A meta-analysis of such studies reported that the bioavailability of Cd in soil increases in the copresence of polyethylene (PE) and polystyrene (PS) MP.^23,24^ Another such study found evidence of altered stress response due to coexposure, reporting elevated phenylpropanoid synthesis and increased linoleic acid metabolism in lettuce.^25^ However, the direct impacts of MNP-Cd coexposure on plant uptake of these pollutants and the mechanisms leading to altered plant uptake of Cd and MNP remain poorly understood.

Thus, the objective of this study was to (1) elucidate the effect of PS NP and Cd on their respective accumulation in hydroponically grown lettuce () coexposed to both of them and (2) gain mechanistic insight into the altered plant uptake of co-occurring contaminants through elemental and metabolomic analysis. Lettuce was selected as a model crop due to its global importance as a leafy vegetable and its frequent use in phytotoxicity studies. It is also one of the EPA-recommended plant species for such studies. A hydroponic system was employed to remove the compounding effects of soil adsorption for Cd and NPs.

## 2 Materials and Methods

### 2.1 Plant Growth Conditions

Lettuce seeds (, cv. Fusion 1) were purchased from Johnny’s Select Seeds (ME, USA). They were sterilized for 10 min using a 2% bleach solution (Clorox, CA, USA) and then rinsed thrice using ultrapure water before germination. The sterilized seeds were sown in batches of 20 on moistened qualitative filter paper in disposable Petri dishes. Germination occurred over 4 days, 2 days under dark conditions and 2 days under a 16:8 day:night cycle at 25 ± 3 °C. Seedlings were then transplanted to foil-wrapped 50 mL falcon tubes (VWR International, PA, USA) filled with 1/4 strength modified Hoagland solution (pH 5.5) purchased from US Biologic (MA, USA). Plants were grown for 28 days under the same light and temperature conditions as for germination, with the hydroponic solution refilled as necessary.

Treatment exposure occurred under the same growth conditions as the initial plant growth. Lettuce seedlings (28-day-old) were removed from the Hoagland solution, and their roots were thoroughly washed with ultrapure water. The seedlings were then transferred to new foil-wrapped tubes containing 50 mL of the respective treatment solutions: CK (1/4 Hoagland solution), Cd (0.85 mg/L cadmium), PS (50 mg/L 500 nm PS NP, Thermo Fisher, MA, USA), and Cd+PS (a combination of PS and Cd treatment at the same concentration). Cd was purchased from Ricca Chemical (TX, USA), while PS NP were purchased from Thermo Fisher (MA, USA).

Treatment concentrations of PS NP and Cd were chosen based on previous reports of measurable impacts on plant metabolism wherein exposure results in detectable plant stress, but not toxicity and plant death.^12,26,27^ Treatment solutions were sonicated at 40,000 Hz for 30 s to ensure homogeneous dispersion of PS NP before plant exposure. Nine plants were prepared for each treatment. Plants received additional 1/4 Hoagland solution every 2 days during the exposure period, with solution additions and final solution volumes recorded to determine total transpiration. At harvest after 7 days of exposure, plants were separated into roots and shoots, with the root portion rinsed with excess ultrapure water to remove residual treatment solution. Tissue storage before analysis after harvest was carried out as stated in the following sections (three replicates per destructive analysis; *n* = 3).

### 2.2 Determination of Photosystem II Efficiency

The chlorophyll fluorescence parameter Fv/Fm was measured to determine the photosystem II (PSII) efficiency immediately prior to harvest. The youngest leaf of each plant, at least 1 cm in width and length, was dark adjusted for 30 min using dark adaptation clips and then analyzed using an OS1p fluorometer (Opti-Sciences, NH, USA).

### 2.3 Determination of Essential Elements and Cd in Plant Tissues

The tissue metal content (Mg, Zn, K, Cu, Fe, and Cd) was determined by inductively coupled plasma–mass spectrometry (ICP-MS, PerkinElmer NexION 300D, MA, USA) following an acid digestion protocol based on EPA Method 200.7. Briefly, plant tissues after harvest were stored in individual brown paper sacks and dried at 65 °C until constant mass was achieved, roughly 24 h. Tissues were weighed, then ground using a mortar and pestle, and transferred to 50 mL polypropylene digestion tubes (VWR International, PA, USA). Four mL of 7 M HNO_3_ and 10 mL of 2 M HCl were added to the tubes; then, the samples were covered with a polypropylene watch glass and digested at 95 °C for 3 h. After digestion, the samples were diluted to 100 mL using an acid-washed volumetric flask. Finally, the samples were analyzed by ICP-MS. A reagent blank and standards using MS-grade metal standards from Ricca Chemical (TX, USA; Table S1) were digested alongside the tissue samples.

### 2.4 Determination of the Nanoplastic Content in Plant Tissues

PS NP in plant tissues were quantified using enzymatic digestion followed by field-emission scanning electron microscopy (FESEM) imaging, following the protocol we developed in a previous study.^28^ Briefly, freshly harvested tissues were stored in 100 mL glass test tubes at −20 °C for 24 h. These tissues were then thawed at room temperature for 30 min. Afterward, 20 mL of 2 g/L macerozyme R-10 solution with 2-(*N*-morpholino)­ethanesulfonic acid buffer (Thermo Fisher, MA, USA, pH 5.0) was added. The mixture was agitated for 24 h at 300 rpm and 37 °C and then was filtered through a Whatman GF-D filter (pore size = 1.2 μm, Whatman, MA, USA) to remove residual plant tissues. The filter was rinsed once with ultrapure water, and the filtrate and rinsate were combined and diluted to 100 mL. A 5 mL portion of the final dilution was filtered through a 0.2 μm nitrocellulose membrane to retain extracted PS NP. The membrane was washed with 10 mL of 50% MeOH and then fully dried in a desiccator before imaging by FESEM (JSM7500, RRID: SCR_022202). The PS NP in the SEM image were used to estimate the PS NP concentration in lettuce tissues via an external standard curve. The external standard curve was obtained by analyzing control plant tissues injected with known volumes of the PS NP stock following the same digestion procedure (Figure S1).

### 2.5 Metabolomics Analysis of Plant Tissues

Water-soluble metabolites in plant tissues were analyzed using liquid chromatography high-resolution/accurate mass spectrometry (LC-HRAM) following extraction with a methanol:chloroform:water-based method.^29^ After harvest, tissues were stored in 50 mL polystyrene falcon tubes at −80 °C overnight, and the lyophilized tissues were homogenized with a bead-based lysis tube (Bertin, MD, USA) on a Precellys 24 tissue homogenizer (Bertin, MD, USA). For extraction, 50 mg of the powdered tissue was transferred to a new lysis tube and extracted twice with 800 μL of ice-cold methanol:chloroform (1:1, v:v) containing 0.25 μg/mL l-leucine as an internal standard. The samples were homogenized for 30 s at a speed of 6000 rpm and then centrifuged at 15,000*g* for 10 min at 4 °C. The supernatants were transferred and combined in a 15 mL polystyrene centrifuge tube on ice. Six hundred μL of ice-cold ultrapure water was added to the extract, which was then vortexed for 30 s and centrifuged at 5000*g* for 10 min at 4 °C. The aqueous supernatant was collected and filtered through a 0.2 μm membrane (Merck Millipore, MA, USA), and again through a 3000 Dat protein concentrator (15,000*g* for 1 h at 4 °C, Thermo Fisher, MA, USA). Prepared samples were finally analyzed by LC-HRAM (full equipment parameters are provided in Text S1).

### 2.6 Statistical Analysis

All data were reported as means (±standard error). Statistical analyses, except where noted, were performed using the R language by applying two-way ANOVA and post hoc Tukey tests where appropriate.^30^ Statistical significance was determined with α = 0.05, with different letters denoting significant differences. Letter case was used to distinguish the comparison of roots and shoots from different treatments. Figures were created using the ggplot2 library.^31^

## 3 Results and Discussion

### 3.1 Treatment Effects on Plant Growth

Cd exposure in the Cd alone treatment group resulted in decreased dry weights of lettuce shoot and root by 28.7 and 40.0%, respectively (Figure 1). This result corresponds to statistically significant decreases in the fresh weight of these tissues by 32.4 and 24.5% (Figure S2). Similar effects were also observed in the joint Cd and PS treatment group, but the PS alone treatment did not cause any significant changes in plant biomass, indicating that Cd exposure is the primary determinant for the observed impact on plant growth by Cd and MNP coexposure. These observations agree with the changes in the plant transpiration rate, which also showed a significant decrease in both the Cd alone and the combined Cd and PS treatment (Figure S3A). The impact of Cd on lettuce growth has been noted in the literature, with significant negative impacts reported in exposure levels as low as 56.2 μg/L.^26^

**Figure 1:**
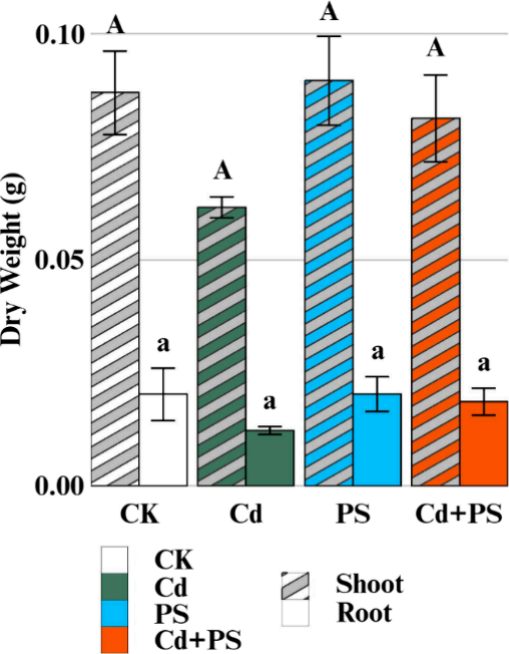
Dry weight of lettuce tissues after 7 days of hydroponic exposure to CK: control with 1/4 Hoagland, Cd: 0.85 mg/L (7.6 μM) cadmium, PS: 50 mg/L of 500 nm polystyrene, and Cd+PS: 50 mg/L of 500 nm polystyrene mixed with 0.85 mg/L cadmium. Solid bars represent plant shoot biomass, and bars with stripes represent plant root biomass. *n* = 3; bars with different letters indicate significant differences (*p* < 0.05).

Past research on Cd phytotoxicity has firmly established that reduced water transpiration is a result of plant physiological responses that alleviate Cd toxicity symptoms. These responses include facilitated vacuolar sequestration and root thickening via increased production of nonwater conducting cortex tissues^32^ and mucilage.^33^ Though they were effective tolerance mechanisms, these protective changes led to reduced transpiration by lettuce in the Cd treatment (Figure S3A). Ultimately, reduced water assimilation could compromise other important physiological processes due to insufficient water availability. No significant impact on PSII activity was observed, likely due to the short duration of this study (Figure S3B). Of interest, though, is the similar reduction in transpiration between the Cd alone and the joint Cd and PS treatments, indicating that the presence of PS NP did not alter this physiological impact of Cd to lettuce plants.

### 3.2 Contents of Essential Elements in Tissues

Mineral contents in the edible tissues of lettuce affect its nutritional value and, thus, were examined. Our results showed that exposure to Cd and MNP did not notably change the concentrations of plant nutrients in lettuce shoots, except for the significantly higher calcium (Ca) in the PS alone treatment as compared to the control and Cd-exposed plants (Figure 2 and Figure S4). The results generally agree with past studies examining single exposure of Cd or PS NP to lettuce and other crops.^25,34^ However, exposure to Cd and PS NP did significantly affect the concentration of Fe and Mg in lettuce roots. For example, exposure to Cd alone significantly increased the root concentration of Fe by 148% compared with the control. Similarly, the root concentration of Mg was increased in all treatment exposures over the control. The Mg concentrations in lettuce root were 59.7, 60.4, and 71.2% higher than the control in Cd alone, PS alone, and joint Cd+PS treatments, respectively. It has been previously shown that plant uptake of Cd is associated with increased activities of ion channels in the actively growing regions of roots, in particular the ion channels specific to Fe^35^ and Mg.^36^ The alteration of Fe and Mg concentrations in lettuce roots suggests that the expression of ion channels might be affected by Cd and PS NP.

**Figure 2:**
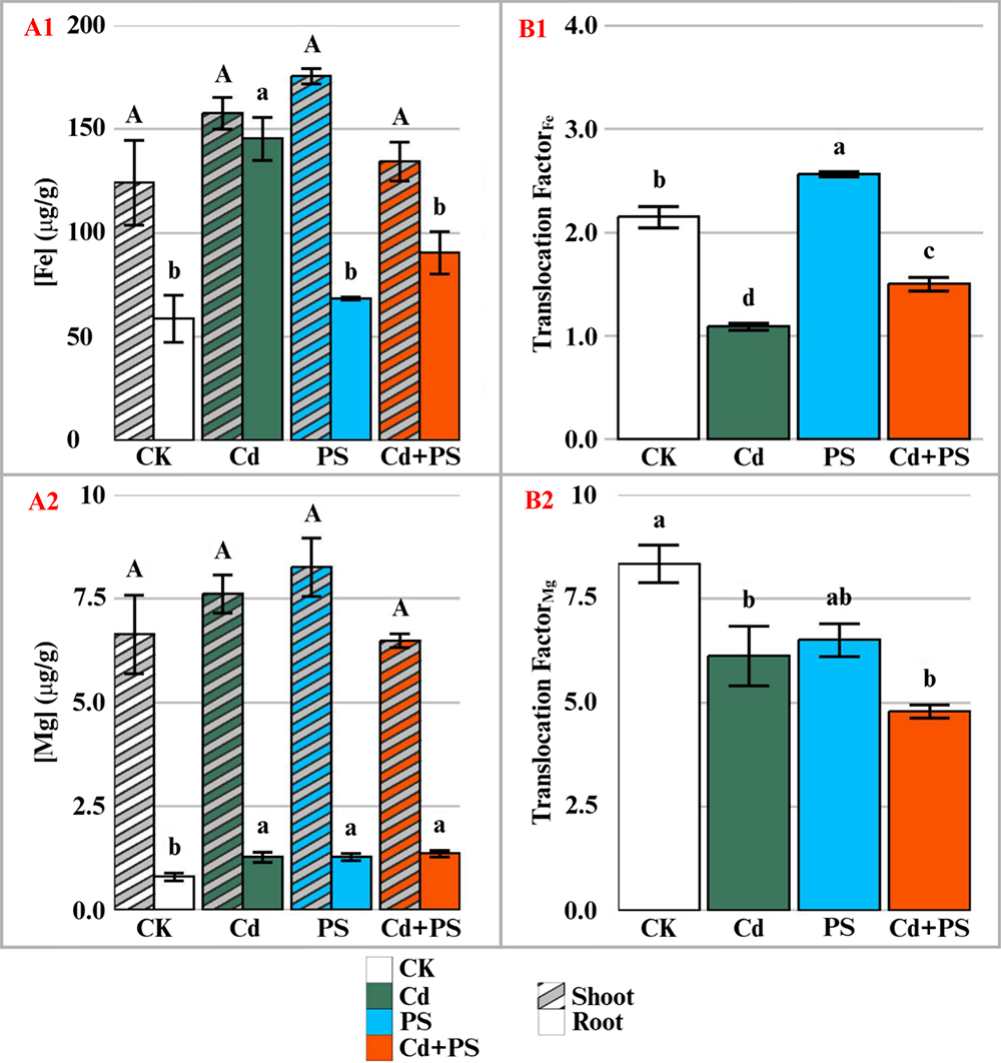
Concentrations in shoot (striped) and root (solid) tissues (A) and translocation factors (B) of iron (**1**) and magnesium (**2**) in lettuce after 7 days of hydroponic exposure to different treatments. CK: control with 1/4 Hoagland, PS: 50 mg/L of 500 nm polystyrene, Cd: 0.85 mg/L cadmium, and Cd+PS: 50 mg/L of 500 nm polystyrene mixed with 0.85 mg/L cadmium. *n* = 3; bars with different letters indicate significant differences (*p* < 0.05).

Of interest, however, is the treatment effect on the translocation of these elements between root and shoot tissues (Figure 2B). PS NP significantly increased the translocation of Fe over the control, but Cd alone and combined Cd and PS NP resulted in significantly decreased translocation of Fe, suggesting that Cd and PS NP had an opposite effect on Fe *in planta* translocation.

### 3.3 Metabolomic Response to Treatment Effects

LC-HRAM analysis, in both positive and negative modes, identified 11,996 compounds. Twenty-seven of them were confidently annotated from the mzCloud and mzVault databases as plant-relevant metabolites and showed significant variations between treatments (Table S2). The identified metabolites were categorized into two major groups. The first group consists of amino acids, dipeptides, and nucleotides, and the second group consists of metabolites directly associated with the plant stress response. The second group can be further divided into metabolites associated with either stress signaling or an oxidative stress response.

The exposure to Cd or PS NP altered the abundance of these metabolites (Figure S5). The most pronounced difference was observed between the joint Cd and PS NP treatment and the control, followed by the Cd alone and the PS alone treatments. Compared to the control, plants exposed to the combined Cd and PS NP treatment had significantly higher reactive oxygen species (ROS) scavenging metabolites, including a diversity of flavonoids. In contrast, lauric acid (LA), an abiotic stress signaling molecule, decreased compared to the control. Exposure to Cd or PS NP alone did not cause significant differences in the abundance of the above-mentioned metabolites from the control, indicating an alternate pathway of stress response to joint Cd and PS NP exposure. However, notable differences were found between the Cd alone treatment and the PS NP alone treatment. The PS NP alone treatment induced a significantly higher abundance of glutathione (GSH), another ROS scavenging species, while many oxidative stress signaling hormones were decreased as compared to the Cd alone treatment. Further, the siderophore and phytochelatin precursor nicotianamine (NAm) had decreased abundance in the PS NP alone treatment as compared to the Cd alone treatment. These results suggest that Cd and PS NP exposure induced distinct oxidative stress responses. Importantly, combined exposure did not lead to a magnification of either of these responses, but rather, a separate stress response pathway appeared to arise, suggesting interdependent reactions to these two cocontaminants.

Hierarchical analysis revealed three distinct clusters of metabolites among treatment groups (Figure 3). The first cluster of metabolites is upregulated in the joint Cd and PS treatment compared to the control. This group of metabolites comprises ROS scavenging species.

**Figure 3:**
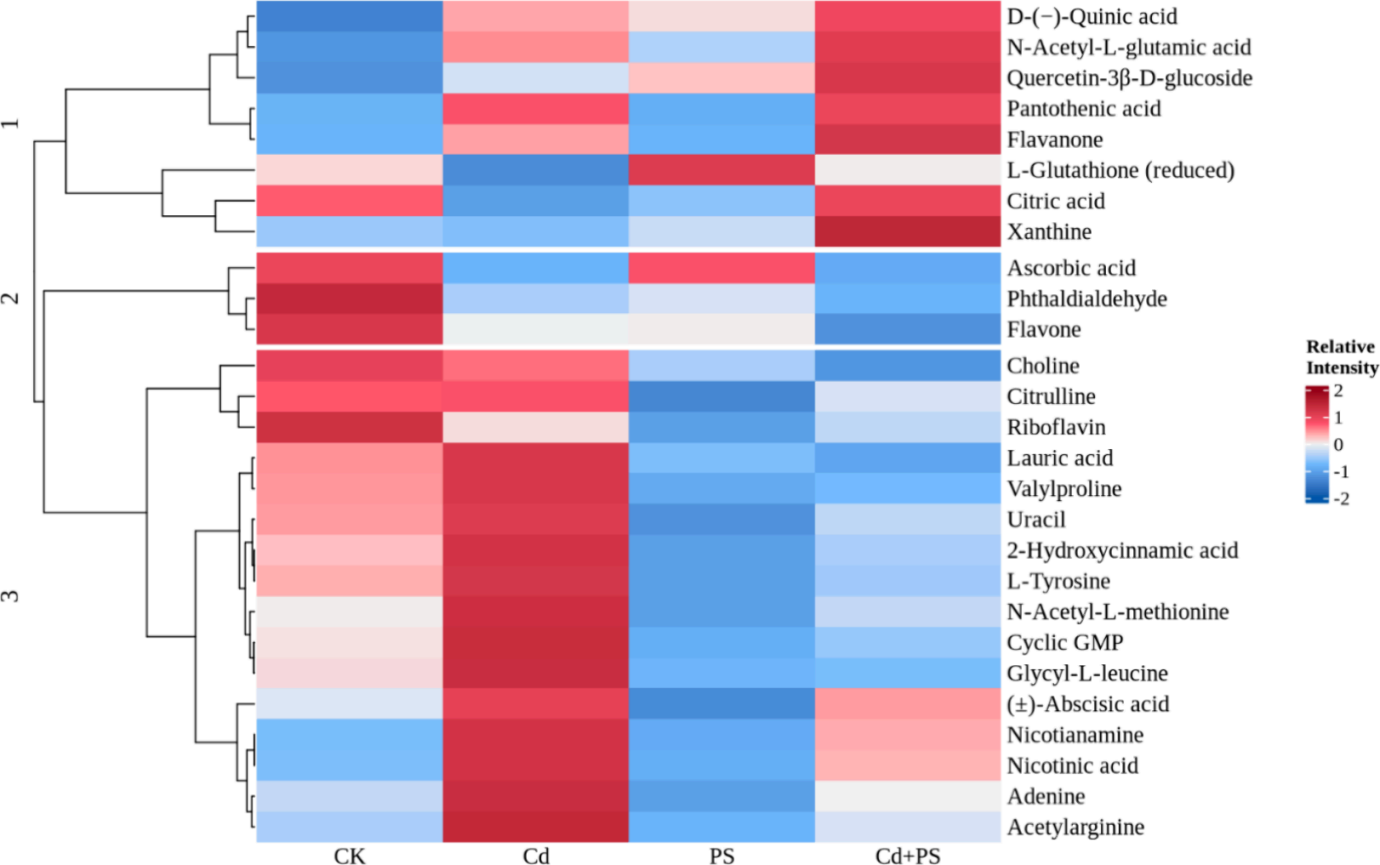
Hierarchical analysis of annotated metabolites showing significant differences between treatments. The color scale indicates the normalized peak area from LC-HRAM for the metabolite labeled on the right-hand side of the figure. Dendrogram developed using Pearson’s correlation between metabolites.

Even though some of these have higher abundance in the Cd alone and PS alone treatments as well, the lack of an apparent additive effect shows how the plant responded to the stress from the joint Cd and PS treatment differently from either contaminant alone.

The second cluster corresponds to metabolites that have lower abundance in each of the exposure treatments compared to that of the control. This group of metabolites likely plays no role in the stress response. Instead, they may represent a metabolic shift from normal to diminished cell function. As energy is diverted toward tolerance mechanisms in the exposure treatments, future growth and productivity may be reduced. The third cluster is composed of signaling molecules, including nicotinic acid (NAR), citrulline, and cyclic guanosine monophosphate (cGMP), as well as precursor metabolites such as NAm and the lignin precursors tyrosine and 2-hydroxycinnamic acid. In this group, metabolites are more abundant in the Cd alone treatment than in the control, while these same compounds in the PS alone and the joint Cd and PS treatments are generally less abundant. The increased presence of these metabolites in the Cd only treatment represents the lettuce’s specific stress response to Cd exposure.

Based on the observed treatment effects on the abundance of these key stress response metabolites, we propose several metabolic pathways that may be differentially affected by Cd and PS NP exposure individually and when they were exposed together (Figure 4). The lettuce response to Cd is consistent with several established pathways: The first is the increased lignin production, as evidenced by the upregulation of 2-hydroxycinnamic acid and tyrosine. Increased lignification provides extracellular sites for Cd adsorption, effectively excluding its entrance to the cell and avoiding damage to sensitive cellular machinery.^22^ The second response of lettuce to Cd exposure is the accumulation of citrulline, possibly resulting in nitric oxide (NO) signaling, a byproduct of arginine oxidation.^37^ This change could consequently lead to the observed upregulation of cGMP, inducing adventitious root formation and stomatal closure.^38^ Combined with the observed upregulation of abscisic acid (AsA), cGMP may also be the cause of the observed reduction in transpiration^39^ (Figure S3A). A secondary impact of NO signaling could be the source of the increased Fe accumulation observed in the lettuce roots of the Cd alone treatment (Figure 2).^40^ In contrast, PS NP exposure resulted in a significant downregulation of these pathways in favor of the upregulation of the ROS scavenger GSH, indicating different coping mechanisms of lettuce with these two types of contaminants (Figure S5).

**Figure 4:**
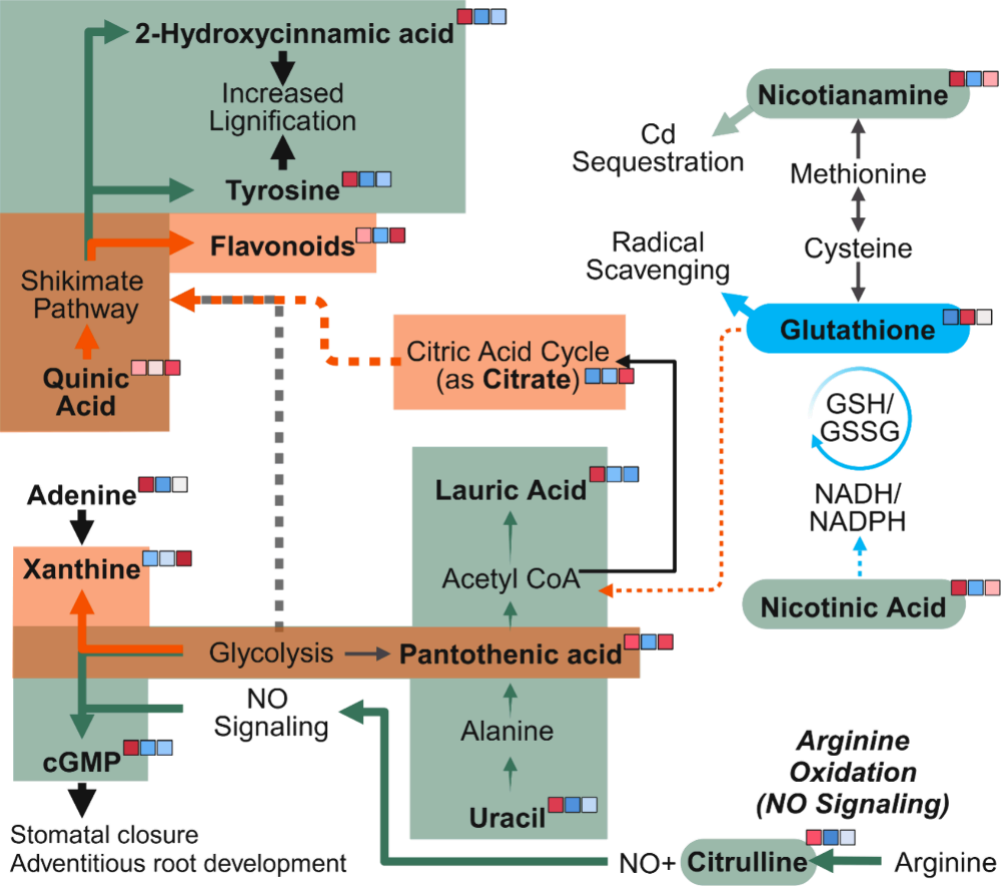
Proposed metabolomic pathways of differentially regulated compounds and their relationship to oxidative stress. Compounds that showed significant differences in regulation between treatments are bolded and annotated with their relative intensities according to the color values in Figure 3 (ordered as Cd, PS, and Cd+PS). Major upregulated pathways are highlighted in green for Cd, light blue for PS, and orange for Cd+PS.

When the lettuce plants are exposed to both contaminants, however, the responses are significantly different from the pathways associated with the individual contaminants. Unlike the PS NP alone treatment, the elevated ROS scavenging compounds come in the form of flavonoids, the production of which is divergent from the lignin pathway upregulated in the Cd alone treatment. Flavonoids play several roles in the plant oxidative stress response. They act as ROS scavengers, mediate ROS scavenger reactivation, and signal for the downregulation of ROS-producing enzymes.^41^ This increased abundance is thus indicative of a long-term stress tolerance mechanism. However, instead of the sequestration and exclusion mechanisms as in the Cd alone treatment, the stress response in the joint Cd and PS NP treatment may result in broad, nonspecific oxidative stress tolerance, highlighting the need to examine the effects of NP and coexisting environmental pollutants. Additionally, cadmium and NP can both significantly alter root anatomy and function. Cd disrupts normal cell division and elongation, leading to reduced root growth, altered vascular development, and increased suberization, which impairs water and nutrient uptake.^42,43^ Similarly, nanoplastics can accumulate in root tissues, inducing oxidative stress, damaging cell structures, and altering root hair development.^44^ When present together, Cd and nanoplastics may have synergistic or additive effects, exacerbating root damage and further compromising plant health and nutrient acquisition.

### 3.4 Plant Uptake of Cadmium and Nanoplastics

PS NP uptake in the PS NP alone treatment agrees with the results of our previous study^28^ and the work performed by Li et al.^45^ wherein root uptake of PS NP is higher, with only slight translocation to shoot tissues after 7 days. In the joint Cd and PS NP treatment group, however, the concentration of PS NP in the shoot tissue significantly increased by 67% over the PS NP alone treatment (Figure 5B). This result is concerning when considering the reduced transpiration observed in the joint Cd and PS NP treatment, indicating a potentially higher concentration of PS NP within the transpiration stream. A longer-term study is needed to assess whether elevated PS NP accumulation in edible tissues would occur over the life cycle of lettuce in the copresence of Cd, compared with PS NP alone exposure.

**Figure 5:**
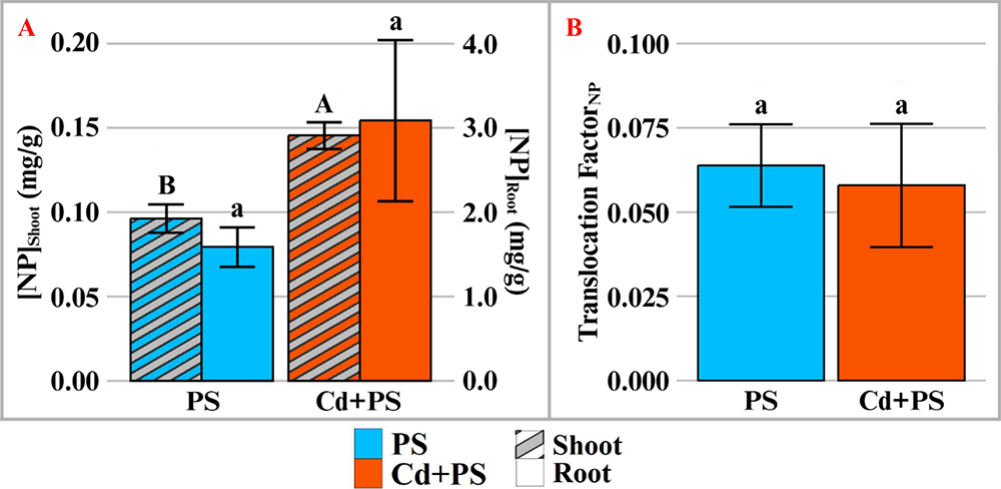
Concentration in shoot (striped) and root (solid) (A) and translocation factor (B) of polystyrene nanoplastics in lettuce after 7 days of hydroponic exposure. PS: 50 mg/L of 500 nm polystyrene; Cd+PS: 50 mg/L of 500 nm polystyrene mixed with 0.85 mg/L cadmium. Values calculated from the standard curve in Figure S1. *n* = 3; bars with different letters indicate significant differences (*p* < 0.05). Note that figure A uses two scales for shoot and root concentrations.

One potential source for this increased uptake of PS NP may be increased adventitious root formation as a result of Cd exposure. The increased adventitious root formation could provide more entry points for PS NP into plants through the theorized root-crack entry pathway.^46^ Further research on the root architectural changes of lettuce in the presence of combined Cd and PS NP is needed to determine if adventitious root formation is responsible for the observed increase in the PS NP concentration in lettuce shoots.

Similar to tissue concentrations of PS NP, lettuce exposed to joint Cd and PS NP showed increased root-to-shoot transport of Cd compared to Cd exposure alone (Figure 6). Even though the Cd root concentration in the joint Cd and PS NP treatment was 69% lower (*p* < 0.05) than that in the Cd alone treatment, the shoot concentration of Cd was 61% higher (*p* > 0.05) (Figure 6B), resulting in a nonsignificant 167% increase of the translocation factor in the joint Cd and PS NP treatment as compared to the Cd alone treatment (Figure 6B).

**Figure 6:**
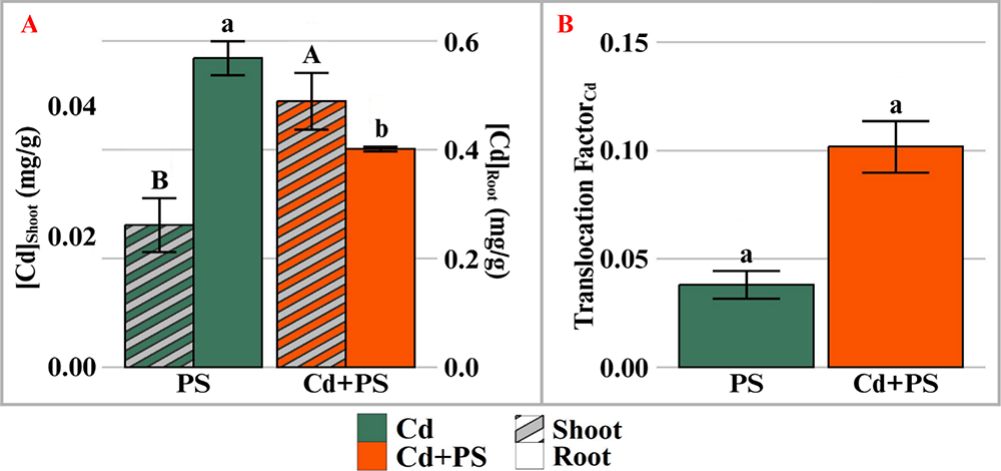
Concentration in shoot (striped) and root (solid) tissues (A) and translocation factor (B) of cadmium in lettuce after 7 days of hydroponic exposure. Cd: 0.85 mg/L cadmium; Cd+PS: 50 mg/L of 500 nm polystyrene mixed with 7.6 μM cadmium. *n* = 3; bars with different letters indicate significant differences (*p* < 0.05). Note that figure A uses two scales for shoot and root concentrations.

These results, combined with the noted changes in the metabolome (Section 3.2), further suggest that the copresence of PS NP with Cd suppressed the sequestration mechanisms that are normally activated in response to lettuce exposure to Cd. This reduced sequestration leads to increased free Cd in the cytosol, where it can participate in ion transport meant for essential elements, such as Fe and Mg, leading to higher accumulation in the shoot tissue of lettuce. This enhanced root-to-shoot translocation was also observed for Fe in the joint Cd and PS NP exposure rather than Cd treatment alone.

These results do not agree with several previous studies, one involving wheat^47^ and another with *Arabidopsis*.^48^ This difference may be explained by the difference in the growth stages of plants used in these different studies: both previous studies used recently germinated seedlings, while the plants used in our study were 28 days old at the time of exposure. It has been previously shown that nutrient assimilation is highest during the vegetative portion of a plant’s growth cycle,^49^ so it may be that the more mature plants in this study were able to successfully combine tolerance mechanisms while still meeting nutritional demands. Further, the size of the PS NP may be an important consideration when discussing the risks of combined Cd and MNP exposure. The joint Cd and PS NP treatment did not induce toxicity symptoms, as measured through the PSII efficiency and harvested biomass. However, a similar study utilizing combined Cd and MP in soil with similarly aged plants observed significant reductions in PSII efficiency and plant biomass.^50^ These discrepancies highlight the need for investigations into the impacts of combined Cd and PS (and other polymer compositions) NP exposure over the whole lifecycle of a plant to fully elucidate their impact on plant health.

While the joint exposure of Cd and PS NP did not appear to aggravate the phytotoxicity of Cd, it poses potential serious risks to consumers in terms of elevated levels of Cd and PS NP in edible tissues. These results and the increasing detection of nanoplastics in agricultural soils call for further study into combined exposure of NPs and cocontaminants. Moving forward, the research community should investigate the coexposure of plants to MNPs and other contaminants in a soil system to determine if these results observed in hydroponic systems remain consistent in a traditional agricultural system. Long-term exposure studies are particularly needed to provide a greater understanding of the effects of coexposure over the whole life cycle of crops and gain more information on their accumulation in the edible tissues such as grains and fruits of other food crops. These efforts should be expanded to other commonly detected MNPs and contaminants in agricultural soils. For plant toxicity investigation, studies on the dose–response relationship for plants exposed to combined contaminants and MNP will shed more light on the potential environmental and health consequences of MNP and environmental cocontaminant exposure.

## Supplementary Material



## Notes

Standard curve for tissue nanoplastic quantification, transpiration response to treatment effects, essential element response to treatment effects, metabolite abundance response to treatment effects, table of metal standards, table of metabolite identification, and summary text of metabolite analysis by LC-MS and identification using Compound Discoverer 3.3 (PDF)
