# Supplementary material for: Synergistic Effects of Polystyrene Nanoplastics and Cadmium on the Metabolic Processes and Their Accumulation in Hydroponically Grown Lettuce ()
Source: J Agric Food Chem. 2025 Jun 24;73(26):16157–64. doi: 10.1021/acs.jafc.5c03215 (PMC12232387; doi:10.1021/acs.jafc.5c03215)
Supplement: Supplementary file 1 [file jf5c03215_si_001.pdf]

Synergistic Effects of Polystyrene Nanoplastics and Cadmium on the Metabolic Processes of and Their Accumulation in Hydroponically Grown Lettuce (*Lactuca sativa*)

Michael Taylor Bryant[1], Lorenzo Rossi[2], Ruipu Mu[3], Zhenyu Cao[1], and Xingmao Ma[1,*]

[1]Department of Civil and Environmental Engineering, Texas A&M University, College Station, TX, 77843

[2]Department of Horticultural Sciences, Texas A&M University, College Station, TX, 77843

[3]Basic Sciences Department, College of Arts and Sciences, University of Health Sciences and Pharmacy in St. Louis, St. Louis, Missouri 63110

***Corresponding Author:**

Dr. Xingmao Ma

Email: xma@civil.tamu.edu

Supplementary Figures: 5

Supplementary Tables: 2

Supplementary Texts: 1

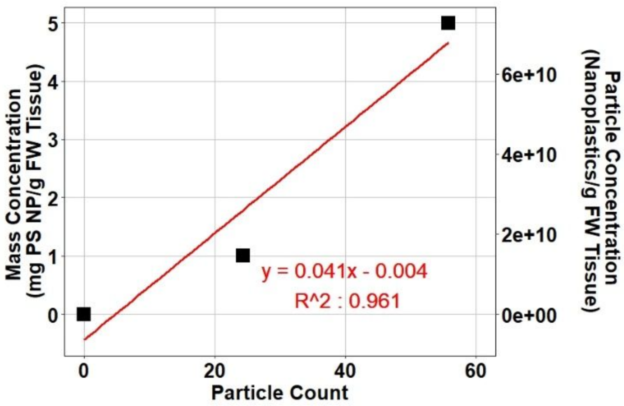

Figure S1: Standard curve used to determine nanoplastic concentration in plant tissues.

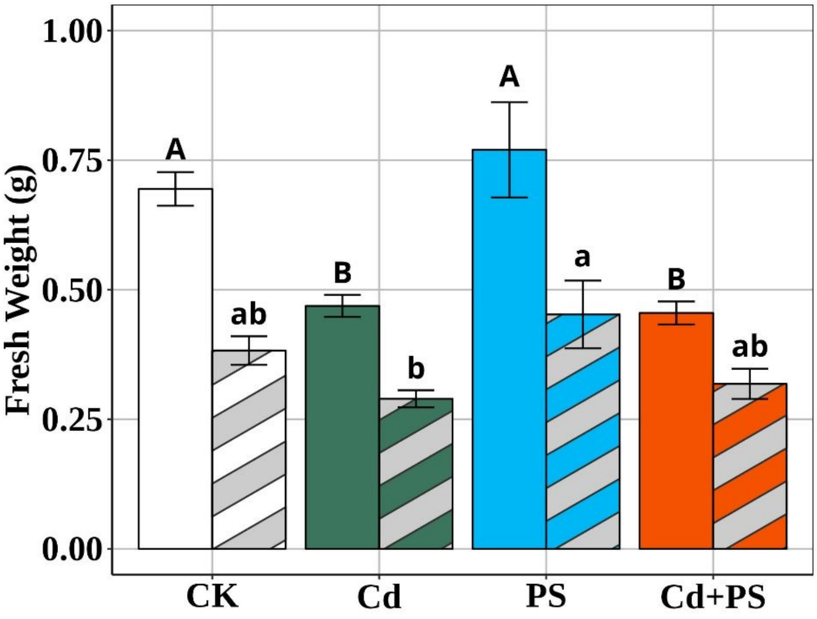

Figure S2: Fresh weight of lettuce tissues after 7 days hydroponic exposure to CK: Control with ¼ Hoagland, Cd: 0.85 mg/L (7.6 μM) cadmium, PS: 50 mg/L of 500 nm Polystyrene, Cd+PS: 50 mg/L of 500 nm Polystyrene mixed with 0.85 mg/L cadmium. Solid bars represent plant shoot biomass and bars with stripes represent plant root biomass. $n = 9$, bars with different letters indicate significant differences ($p < 0.05$).

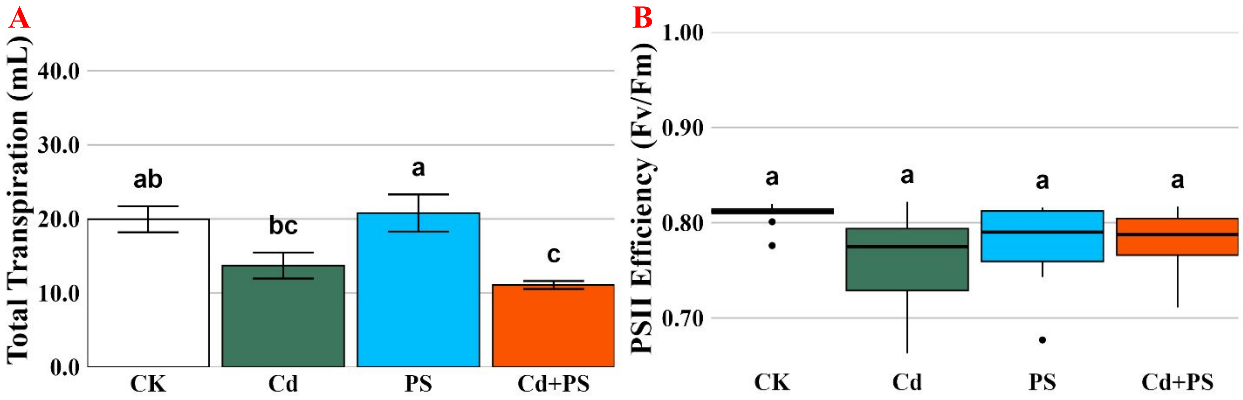

Figure S3: Total transpiration (**A**) and PSII efficiency (**B**). CK: Control with ¼ Hoagland, Cd: 7.6 µM cadmium, PS: 50 mg/L of 500 nm Polystyrene, Cd+PS: 50 mg/L of 500 nm Polystyrene mixed with 7.6 µM cadmium. n = 9, bars with different letters indicate significant differences ($p < 0.05$).

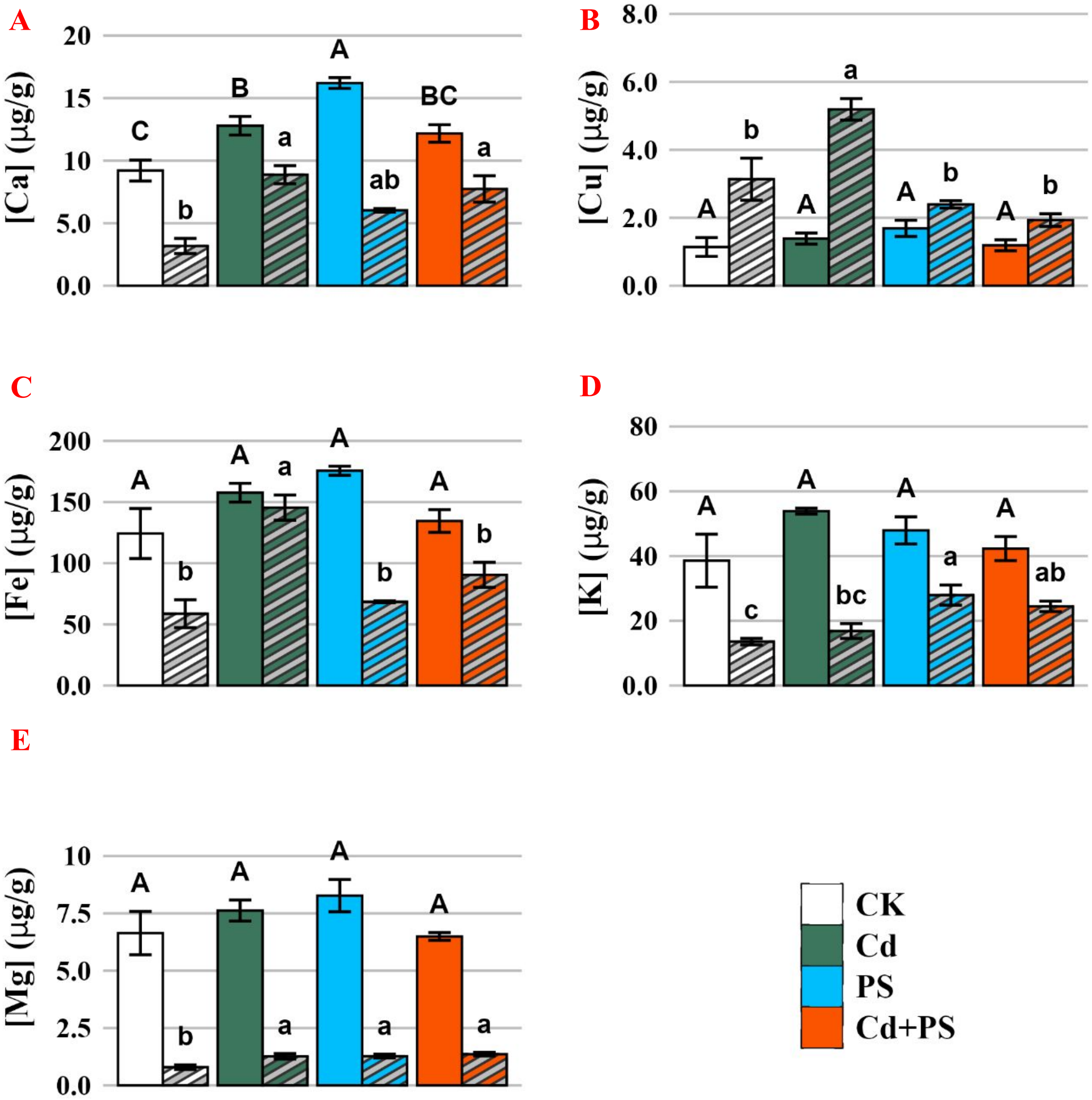

Figure S4: Shoot and root calcium (**C**), copper (**B**), iron (**C**), potassium (**D**), and magnesium (**E**) concentrations in lettuce tissues after 7 days of hydroponic exposure. Solid bars represent plant shoot biomass and bars with stripes represent plant root biomass. CK: Control with ¼ Hoagland, PS: 50 mg/L of 500 nm Polystyrene, Cd: 7.6 µM cadmium, Cd+PS: 50 mg/L of 500 nm Polystyrene mixed with 7.6 µM cadmium. n = 3, letters indicate significant differences ($p < 0.05$).

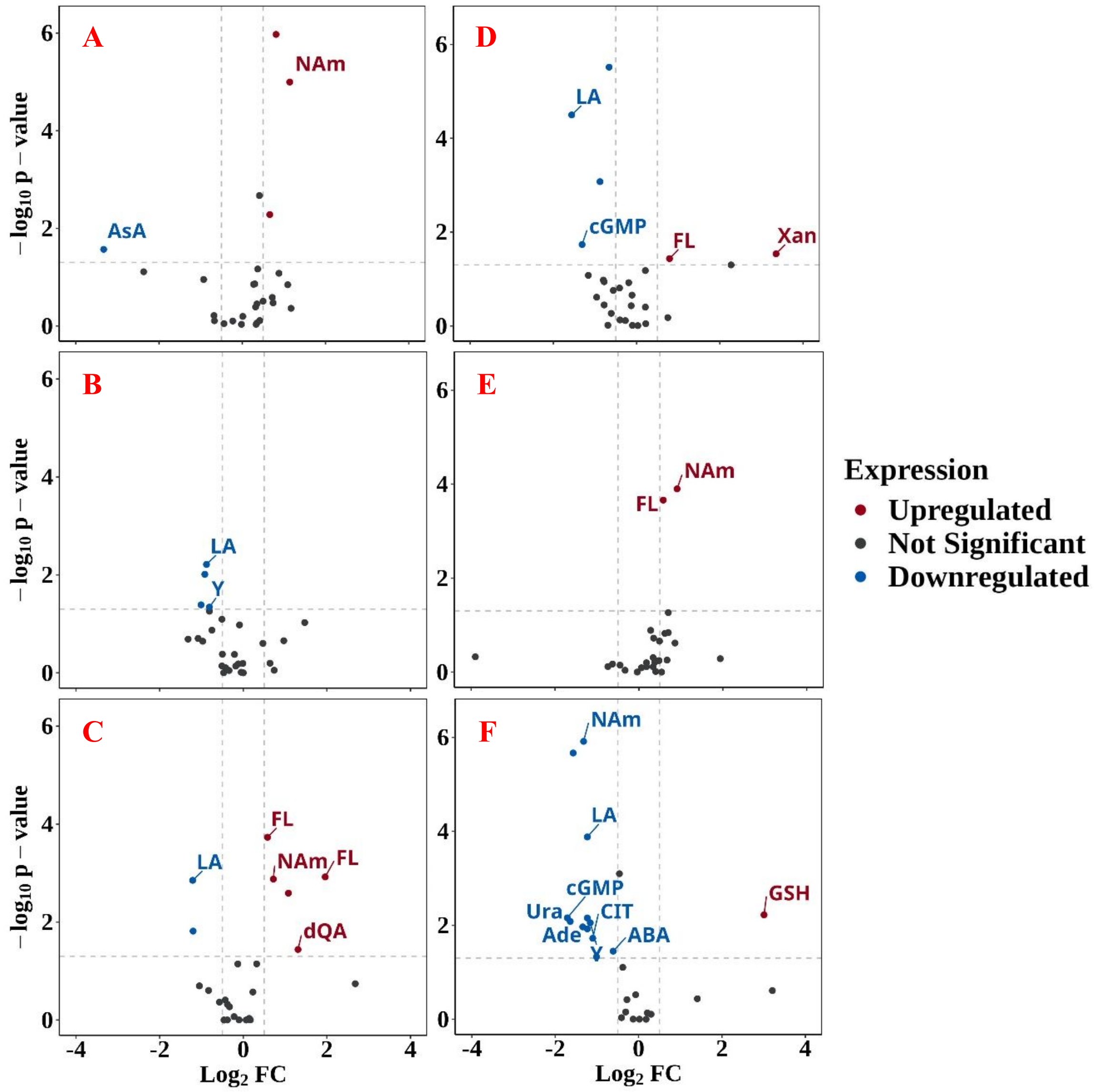

Figure S5: Volcano plots comparing metabolites generated from positive and negative ionization mode. Comparisons are (**A**) Cd versus CK, (**B**) PS versus CK, (**C**) Cd+PS versus CK, (**D**) Cd+PS versus Cd, (**E**) Cd+PS versus PS, and (**F**) PS versus Cd exposed plants. Upregulated metabolites are indicated in red, downregulated in blue with significant compounds determined as having a Log_2 Fold Change increase greater than 0.5 and $\alpha = 0.05$. Unlabeled significant compounds correspond to dipeptide species.

Table S1: Metal standards used to determine associated content in tissues via ICP-MS. Values in mg/L.

Metal	Std 1	Std 2	Std 3
Ca	0.25	0.75	1.5
Mg	0.1	0.25	0.5
K	2.5	5	10
Fe	0.005	0.1	0.025
Cu	0.0005	0.001	0.0025
Cd	1	5	10

Table S2: Annotation source and LC/MS results of metabolites showing significant differences in abundance between treatments

Metabolite	Annotation Source	m/z	Retention time (min)
D-(-)-Quinic acid	mzCloud	191.05543	0.57
N-Acetyl-L-glutamic acid	Metabolika	188.0558	2.567
Quercetin-3β-D-glucoside	mzCloud	463.08874	7.191
Pantothenic acid	mzCloud	218.10306	3.159
Flavanone	mzCloud	225.09116	6.813
L-Glutathione	mzCloud	308.09119	1.218
Citric acid	mzCloud	191.01904	1.903
Xanthine	mzCloud	153.04097	2.058
Ascorbic acid	mzCloud	175.024	1.243
Phthaldialdehyde	mzCloud	135.0443	6.063
Flavone	mzCloud	223.07556	10.865
Choline	mzCloud	104.10756	1.078
Citrulline	mzVault	174.08757	1.114
Riboflavin	mzVault	377.07556	6.357
Lauric acid	mzCloud	199.16971	9.301
Valylproline	mzCloud	215.13934	2.621
Uracil	mzCloud	113.03501	1.697
2-Hydroxycinnamic acid	mzCloud	165.05486	1.312
L-Tyrosine	mzCloud	182.08142	1.31
N-Acetyl-L-methionine	mzCloud	217.1297	1.151
Cyclic GMP	mzVault	344.04045	2.728
Glycyl-L-leucine	mzCloud	189.12361	2.299
(±)-Abscisic acid	mzCloud	265.14346	4.847
Nicotianamine	mzVault	245.13586	1.07
Nicotinic acid	mzCloud	124.03962	1.202
Adenine	mzCloud	136.06198	1.196
Acetylarginine	mzCloud	217.1297	1.151

Text S1: LC-MS Sample Analysis

Untargeted liquid chromatography high resolution accurate mass spectrometry (LC-HRAM) analysis was performed on a Q Exactive Plus orbitrap mass spectrometer (Thermo Scientific, Waltham, MA) coupled to a binary pump HPLC (UltiMate 3000, Thermo Scientific). Full MS spectra were obtained at 70,000 resolution (200 m/z) with a scan range of 50–750 m/z. Full MS followed by ddMS2 scans were obtained at 35,000 resolution (MS1) and 17,500 resolution (MS2). Samples were maintained at 4 °C before injection. The injection volume was 10 µL. Chromatographic separation was achieved on a Synergi Fusion 4µm, 150 mm x 2 mm reverse phase column (Phenomenex, Torrance, CA) maintained at 30 °C using a solvent gradient method. Solvent A was water (0.1% formic acid). Solvent B was methanol (0.1% formic acid). The gradient method used was 0-5 min (10% B to 40% B), 5-7 min (40% B to 95% B), 7-9 min (95% B), 9-9.1 min (95% B to 10% B), 9.1-13 min (10% B). The flow rate was 0.4 mL min^{-1}. Sample acquisition was performed Xcalibur (Thermo Scientific). Data analysis was performed with Compound Discoverer 3.3 (Thermo Scientific).
